# A complex regulatory network governs the expression of symbiotic genes in *Sinorhizobium fredii* HH103

**DOI:** 10.3389/fpls.2023.1322435

**Published:** 2023-12-21

**Authors:** Pilar Navarro-Gómez, Francisco Fuentes-Romero, Francisco Pérez-Montaño, Irene Jiménez-Guerrero, Cynthia Alías-Villegas, Paula Ayala-García, Andrés Almozara, Carlos Medina, Francisco-Javier Ollero, Miguel-Ángel Rodríguez-Carvajal, José-Enrique Ruiz-Sainz, Francisco-Javier López-Baena, José-María Vinardell, Sebastián Acosta-Jurado

**Affiliations:** ^1^ Departamento de Microbiología, Universidad de Sevilla, Sevilla, Spain; ^2^ Departamento de Biología Molecular e Ingeniería Bioquímica, Centro Andaluz de Biología del Desarrollo, Universidad Pablo de Olavide/Consejo Superior de Investigaciones Científicas/Junta de Andalucía, Sevilla, Spain; ^3^ Departamento de Química Orgánica, Facultad de Química, Universidad de Sevilla, Sevilla, Spain

**Keywords:** rhizobium-legume symbiosis, Nod factors, *NodD1*, *NodD2*, *NolR*, *SyrM*, *TtsI*, symbiotic genes regulatory network

## Abstract

**Introduction:**

The establishment of the rhizobium-legume nitrogen-fixing symbiosis relies on the interchange of molecular signals between the two symbionts. We have previously studied by RNA-seq the effect of the symbiotic regulators NodD1, SyrM, and TtsI on the expression of the symbiotic genes (the *nod* regulon) of *Sinorhizobium fredii* HH103 upon treatment with the isoflavone genistein. In this work we have further investigated this regulatory network by incorporating new RNA-seq data of HH103 mutants in two other regulatory genes, *nodD2* and *nolR*. Both genes code for global regulators with a predominant repressor effect on the *nod* regulon, although NodD2 acts as an activator of a small number of HH103 symbiotic genes.

**Methods:**

By combining RNA-seq data, qPCR experiments, and b-galactosidase assays of HH103 mutants harbouring a *lacZ* gene inserted into a regulatory gene, we have analysed the regulatory relations between the *nodD1*, *nodD2*, *nolR*, *syrM*, and *ttsI* genes, confirming previous data and discovering previously unknown relations.

**Results and discussion:**

Previously we showed that HH103 mutants in the *nodD2*, *nolR*, *syrM*, or *ttsI* genes gain effective nodulation with *Lotus japonicus*, a model legume, although with different symbiotic performances. Here we show that the combinations of mutations in these genes led, in most cases, to a decrease in symbiotic effectiveness, although all of them retained the ability to induce the formation of nitrogen-fixing nodules. In fact, the *nodD2*, *nolR*, and *syrM* single and double mutants share a set of Nod factors, either overproduced by them or not generated by the wild-type strain, that might be responsible for gaining effective nodulation with *L. japonicus*.

## 1 Introduction

Rhizobia are soil proteobacteria able to establish a symbiotic nitrogen-fixing interaction with legumes (Poole et al., 2018). These bacteria infect legume roots and induce the formation of new root organs called nodules. Later on, rhizobia colonize intracellularly these nodules and differentiate into bacteroids able to fix N_2_. Bacteroids supply combined nitrogen to the plant and, in return, are fed with C and energy sources. The complete set of events that takes place in this symbiotic interaction is known as the nodulation process (Roy et al., 2020; Yang et al., 2022).

The nodulation process relies on a complex molecular dialogue established between both partners (Peck et al., 2013; López-Baena et al., 2016). The first step is exudation of flavonoids by plant roots. The interaction of appropriate flavonoids with the rhizobial protein NodD, which belongs to the LysR family of transcriptional regulators, will affect the expression of many bacterial genes related to symbiosis. Flavonoid-NodD complexes bind to conserved DNA sequences called *nod* boxes (NBs) that are located upstream of many rhizobial symbiotic genes. One of the set of genes regulated by NodD and flavonoids is that involved in the production and secretion of specific molecular signals called Nod factors (NFs). NFs are N-acetyl-glucosamine oligosaccharides harbouring different decorations, and each rhizobial strain produces a specific set of NFs (Ghantasala and Choudhury, 2022). These bacterial signals are perceived by LysM receptors located in the root hairs membrane. If NFs are compatible, this recognition event triggers bacterial infection and nodule organogenesis (Roy et al., 2020; Jhu and Oldroyd, 2023). Thus, the flavonoid/NodD and NF/LysM receptor interactions are key events for the establishment of the symbiotic interaction.

NFs are not the only rhizobial molecular signal involved in symbiosis. In some rhizobial strains, such as most of those belonging to the genus *Bradyrhizobium* spp. or to *Sinorhizobium fredii*, NodD and flavonoids also activate the production of effector proteins that are secreted into the cytoplasm of host cells by a type 3 secretion system (T3SS) (Jiménez-Guerrero et al., 2022; Teulet et al., 2022). This is because the expression of the transcriptional regulator TtsI is also activated by NodD and flavonoids. TtsI interacts with conserved DNA sequences called *tts* boxes (TBs) triggering the expression of genes coding both for the symbiotic type 3 secretion system (T3SS) machinery and for effector proteins (called T3Es). T3Es might alter host pathways or suppress host defense responses, with different effects in the symbiotic process (positive, neutral, negative) depending on the specific rhizobium-legume couple and, in some specific cases, can promote symbiosis even in the absence of NFs (Jiménez-Guerrero et al., 2022; Teulet et al., 2022). In addition to NFs and T3Es, various bacterial surface polysaccharides, such as exopolysaccharides (EPS), lipopolysaccharides (LPS), K-antigen capsular polysaccharides (KPS), and cyclic glucans (CG), may have significant roles in symbiosis. They can function as crucial molecules required for the progression of the infection and/or suppress plant defense responses (Downie, 2010; López-Baena et al., 2016; Acosta-Jurado et al., 2021).

*Sinorhizobium fredii* HH103 Rif^R^ (hereafter referred to as HH103) is a broad host-range rhizobial strain that nodulates many different legumes, including different species of the *Glycine* genus such as *G. soja* and *G. max*, its natural host plants (Margaret et al., 2011; Vinardell et al., 2015). In this strain, different RNA-seq studies have been performed to analyse the regulation of the expression of bacterial genes that might be involved in symbiosis with legumes. These studies have been carried out either in the presence of effective *nod* gene inducers such as genistein or in the presence of *Lotus japonicus* root exudates (Vinardell et al., 2004a; Pérez-Montaño et al., 2016; Acosta-Jurado et al., 2019; Acosta-Jurado et al., 2020). These studies showed that one hundred HH103 genes (the so-called *nod* regulon) respond to the presence of genistein and that NodD1 is the main positive regulator of the HH103 *nod* regulon. NodD1 activates the expression of those genes related to NFs and T3Es production as well as that of different secondary transcriptional regulators such as NodD2, SyrM, and TtsI (Pérez-Montaño et al., 2016). These studies also demonstrated that TtsI is a positive regulator responsible for the genistein-dependent induction of 35 genes involved in T3SS assembly and T3Es production. Additional RNA-seq studies showed that SyrM is a global regulator that affects the expression of 279 genes in the presence of genistein. The effect of SyrM on the *nod* regulon is variable, since this protein acts as a repressor of a number of genes (including those related to NFs production) but as an activator of others (such as *nodD2*, and genes putatively related to indole-3-acetic acid synthesis and nitrogen fixation) (Acosta-Jurado et al., 2020). Other RNA-seq studies, performed in the presence of *L. japonicus* root exudates, have analysed the role of NodD2 and the global regulator NolR in HH103 (Acosta-Jurado et al., 2019). These studies showed that the absence of each of these proteins affected the expression of hundreds of genes indicating that not only NolR but also NodD2 is a global regulator in HH103. Regarding the *nod* regulon, NodD2 and NolR appeared to function as repressors of genes related to NFs production and the T3SS. However, these studies also showed that NodD2 acts as an activator of several genes belonging to this regulon. Some of the genes induced by NodD2 upon treatment with *L. japonicus* root exudates are also induced by SyrM in the presence of genistein, such as the previously mentioned genes putatively related to indole-3-acetic acid synthesis and nitrogen fixation.

HH103 NodD1 is essential for symbiosis with all the host-legumes tested so far (Margaret et al., 2011; López-Baena et al., 2016). However, the symbiotic relevance of NodD2, NolR, SyrM, and TtsI, which play important roles in the fine-tune modulation of the expression of the *nod* regulon, is variable. The lack of NodD2, NolR, SyrM, or TtsI provokes partial impairment in symbiosis with soybean (Vinardell et al., 2004b; López-Baena et al., 2008);. However, mutation of either *nodD2*, *nolR*, or *syrM* allows effective nodulation on two legumes in which wild-type HH103 only induces the formation of non-colonized ineffective nodules: the model legume *Lotus japonicus* and *Phaseolus vulgaris* (Acosta-Jurado et al., 2016a; Acosta-Jurado et al., 2019; Acosta-Jurado et al., 2020; Fuentes-Romero et al., 2022). This positive effect might be due to the increased NFs production observed in all these mutants. Interestingly, mutants in *ttsI* also gain effective nodulation in *L. japonicus* but not in *P. vulgaris* (Jiménez-Guerrero et al., 2020; Fuentes-Romero et al., 2022), indicating that some of the T3Es produced by HH103 block nodulation with the former plant.

In this work we have further investigated the complex regulatory network that governs the expression of the HH103 *nod* regulon. Thus, we have completed our analyses of the effect of genistein on HH103 gene expression by carrying out RNA-seq experiments in the HH103 *nolR* and *nodD2* mutant backgrounds. We have investigated the putative regulatory connections among the main regulatory genes of the *nod* regulon: *nodD1*, *nodD2*, *nolR*, *syrM*, and *ttsI*. In addition, we have studied the symbiotic abilities of HH103 *ttsI* mutant derivatives carrying an additional mutation in either *nodD2*, *nolR*, or *syrM*, as well as the production of NFs in the three possible combinations of HH103 double mutants in the *nodD2*, *nolR*, and *syrM* genes.

## 2 Materials and methods

### 2.1 Basic molecular and microbiological techniques

**Table 1** contains all the bacterial strains and plasmids employed in this work. *Sinorhizobium fredii* strains were grown at 28 °C on TY medium (Beringer, 1974) or in yeast extract/mannitol (YM) medium (Vincent, 1970). *Escherichia coli* was cultured on LB medium (Sambrook and Russell, 2001) at 37 °C. Supplementation with antibiotics and/or genistein, when necessary, was carried out as described by Vinardell et al. (2004a).

**Table 1:** Bacterial strains and plasmids used in this work.

Strain	Derivation and relevant properties^a^	Source or reference
*Sinorhizobium fredii*		
HH103 Rif^R^ (=SVQ269)	Spontaneous Rif^R^ derivative of HH103	Madinabeitia et al., 2002
SVQ318	HH103 Rif^R^ *nodD1::*Ω	Vinardell et al., 2004a
SVQ515	HH103 Rif^R^ *nodD2::*Ω	López-Baena et al., 2008
SVQ533	HH103 Rif^R^ *ttsI::*Ω	López-Baena et al., 2008
SVQ534	HH103 Rif^R^ *ttsI*::*lacZ*Δp-Gm^R^	López-Baena et al., 2008
SVQ544	HH103 Rif^R^ *nodD1::*Ω *ttsI*::*lacZ*Δp-Gm^R^	López-Baena et al., 2008
SVQ545	HH103 Rif^R^ *nodD2::*Ω *ttsI*::*lacZ*Δp-Gm^R^	López-Baena et al., 2008
SVQ548	HH103 Rif^R^ *nolR*::*lacZ*Δp-Gm^R^	Acosta-Jurado et al., 2016b
SVQ549	HH103 Rif^R^ *nodD1::*Ω *nolR*::*lacZ*Δp-Gm^R^	López-Baena et al., 2008
SVQ550	HH103 Rif^R^ *ttsI::*Ω *nolR*::*lacZ*Δp-Gm^R^	López-Baena et al., 2008
SVQ551	HH103 Rif^R^ *nodD2::*Ω *nolR*::*lacZ*Δp-Gm^R^	López-Baena et al., 2008
SVQ553	HH103 Rif^R^ *nolR::*Ω *ttsI*::*lacZ*Δp-Gm^R^	López-Baena et al., 2008
SVQ555	HH103 Rif^R^ *nodD1::*Ω n*odD2*::*lacZ*Δp-Gm^R^	Acosta-Jurado et al., 2019
SVQ556	HH103 Rif^R^ *ttsI::*Ω n*odD2*::*lacZ*Δp-Gm^R^	López-Baena et al., 2008
SVQ557	HH103 Rif^R^ *nolR::*Ω	López-Baena, F.J.
SVQ724	HH103 Rif^R^ Δ*syrM*	Acosta-Jurado et al., 2019
SVQ727	HH103 Rif^R^ *syrM*::*lacZ*Δp-Gm^R^	Acosta-Jurado et al., 2020
SVQ770	HH103 Rif^R^ Δ*syrM nodD2*::*lacZ*Δp-Gm^R^	This work
SVQ786	HH103 Rif^R^ *nodD1*::*lacZ*Δp-Gm^R^	This work
SVQ787	HH103 Rif^R^ *nodD2*::*lacZ*Δp-Gm^R^	This work
SVQ788	HH103 Rif^R^ *nolR::*Ω *nodD1*::*lacZ*Δp-Gm^R^	This work
SVQ789	HH103 Rif^R^ Δ*syrM nolR*::*lacZ*Δp-Gm^R^	This work
SVQ811	HH103 Rif^R^ Δ*syrM nodD1*::*lacZ*Δp-Gm^R^	This work
SVQ814	HH103 Rif^R^ *nodD1::*Ω *syrM*::*lacZ*Δp-Gm^R^	This work
SVQ817	HH103 Rif^R^ *ttsI::*Ω *nodD1*::*lacZ*Δp-Gm^R^	This work
SVQ819	HH103 Rif^R^ *nodD2::*Ω *syrM*::*lacZ*Δp-Gm^R^	This work
SVQ828	HH103 Rif^R^ *ttsI*::*lacZ*Δp-Gm^R^ *syrM*::pK18*mob*	This work
SVQ836	HH103 Rif^R^ *nodD2::*Ω *nodD1*::*lacZ*Δp-Gm^R^	This work
SVQ837	HH103 Rif^R^ *nolR::*Ω *nodD2*::*lacZ*Δp-Gm^R^	This work
SVQ842	HH103 Rif^R^ *ttsI::*Ω *syrM*::*lacZ*Δp-Gm^R^	This work
SVQ843	HH103 Rif^R^ *nolR::*Ω *syrM*::*lacZ*Δp-Gm^R^	This work
*Escherichia coli*		
DH5α	*supE44* Δ*lacU169 hsdR17 racA1 endA1 gyr96 thi-1 relA1* Nx^R^	Stratagene
BTH101	*cya-99, araD139, galE15, galK16, rpsL1 (*Str^R^ *), hsdR2, mcrA1, mcrB1*	Euromedex, TwoHybrid (BACTH) System Kit
S17-1	*pro, res- hsdR17 (rK- mK+) recA-* with an integrated *RP4-2-Tc::Mu-Km::Tn7*, Tp^R^	Simon, 1984
Plasmids		
pAB2001	Ap^R^ resistant vector containing the *lacZ*Δp-Gm^R^ cassette	Becker et al., 1995
pBluescript II SK+	Cloning and sequencing vector, Ap^R^	Stratagene
pGEM-T-Easy	Cloning vector for PCR amplified fragments	Promega
pRK2013	Helper plasmid, Km^R^	Figurski and Helinski, 1979
pBBR1MCS-2	Broad host-range cloning vector, Km^R^	Kovach et al., 1995
pK18*mob*	Cloning vector, *sacB* gene, Km^R^, suicide in rhizobia	Schäfer et al., 1994
pKT25	*B. pertussis cya* T25‐expression plasmid, Km^R^	Euromedex, TwoHybrid (BACTH) System Kit
pKNT25	*B. pertussis cya* NT25‐expression plasmid, Km^R^	Euromedex, TwoHybrid (BACTH) System Kit
pKT25-zip	*B. pertussis cya* T25‐leucine zipper fusion, Km^R^	Euromedex, TwoHybrid (BACTH) System Kit
pUT18	*B. pertussis cya* T18‐expression plasmid, Km^R^	Euromedex, TwoHybrid (BACTH) System Kit
pUT18C	*B. pertussis cya* T18C‐expression plasmid, Km^R^	Euromedex, TwoHybrid (BACTH) System Kit
pUT18C-zip	*B. pertussis cya* T18C‐leucine zipper fusion, Km^R^	Euromedex, TwoHybrid (BACTH) System Kit
pMUS296	pMP92 carrying a ~1,7 kb *EcoR*V-*Apa*I fragment containing the *nodD1* gene	Vinardell et al., 2004a
pMUS534	pK18*mob* carrying a 6,0 kb *Hind*III fragment containing the *nodD1*::*lacZ*Δp-Gm^R^ fusion	Vinardell et al., 2004a
pMUS672	pBluescript carrying a 2,547-bp *Eco*RI fragment containing HH103 *nolR*	Vinardell et al., 2004b
pMUS675	pMP92 carrying a ~2,5 kb *EcoR*I fragment containing the *nolR* gene	Vinardell et al., 2004b
pMUS741	pMP92 carrying a ~1,4 kb *EcoR*I fragment containing the *ttsI* gene and its *nod* box	López-Baena et al., 2008
pMUS746	pMP92 carrying a ~1,4 kb *EcoR*I fragment containing the *nodD2* gene	López-Baena et al., 2008
pMUS788	pGEM-T-Easy derivative carrying the *lacZ*Δp-Gm^R^ as a 4.3 kb *Sma*I fragment from pAB2001 into *nodD2*	Acosta-Jurado et al., 2019
pMUS789	pK18*mob* carrying a ~5,7 kb *EcoR*I fragment containing the *nodD2*::*lacZ*Δp-Gm^R^ fusion	This work
pMUS857	pMUS672 containing the *lacZ*Δp-Gm^R^ into the *Nco*I site of *nolR*	This work
pMUS859	pK18*mob* carrying a 7,0 kb *EcoR*I fragment containing the *nolR*::*lacZ*Δp-Gm^R^ fusion	This work
pMUS1232	pK18*mobsac* carrying a 1,5 kb *Hind*III- *BamH*I fragment containing the deleted version of the *syrM* gene	Acosta-Jurado et al., 2020
pMUS1234	pK18*mob* carrying a 2,0 kb *Hind*III- *BamH*I containing the *syrM*::*lacZ*Δp-Gm^R^ fusion	Acosta-Jurado et al., 2020
pMUS1447	pKT25 carrying a 1,0 kb *Kpn*I-*Xba*I fragment containing the *syrM* gene	This work
pMUS1449	pKT25 carrying a 1,0 kb *Kpn*I-*Xba*I fragment containing the *nodD1* gene	This work
pMUS1450	pKNT25 carrying a 1,0 kb *Kpn*I-*Xba*I fragment containing the *nodD1* gene	This work
pMUS1451	pUT18C carrying a 1,0 kb *Kpn*I-*Xba*I fragment containing the *nodD1* gene	This work
pMUS1452	pUT18 carrying a 1,0 kb *Kpn*I-*Xba*I fragment containing the *nodD2* gene	This work
pMUS1453	pUT18C carrying a 1,0 kb *Kpn*I-*Xba*I fragment containing the *nodD2* gene	This work
pMUS1454	pUT18 carrying a 1,0 kb *Kpn*I-*Xba*I fragment containing the *nodD1* gene	This work
pMUS1455	pKT25 carrying a 1,0 kb *Kpn*I-*Xba*I fragment containing the *nodD2* gene	This work
pMUS1457	pKNT25 carrying a 1,0 kb *Kpn*I-*Xba*I fragment containing the *nodD2* gene	This work
pMUS1492	pK18*mob* carrying a *syrM* ~0,6kb *EcoR*I- *Xba*I internal fragment	This work
pMUS1514	pBBR1MCS-2 carrying a ~1,9 kb *Kpn*I-*Xba*I fragment containing the *syrM* gene	This work

Recombinant DNA techniques were performed as described by Sambrook and Russell (2001). Specific details about DNA-DNA hybridization and PCR amplifications can be found in previous works of our group (Vinardell et al., 2004b; Pérez-Montaño et al., 2016). For quantitative PCR (*q*PCR) experiments, total RNA (DNA free), obtained by using the High Pure RNA Isolation Kit (Roche) and the RNAase Free DNAse (Qiagen), was reverse transcribed to cDNA with the QuantiTec Reverse Transcription Kit (Qiagen). Quantitative PCR (*q*PCR) experiments were conducted as described by Pérez-Montaño et al. (2016). Normalization was carried out with the *S. fredii* HH103 RNA 16S gene. For each treatment, the fold changes showed in this work have been obtained by performing at least two independent experiments with three technical replicates and using the ΔΔCt method (Pfaffl et al., 2002). **Table 2** summarizes all the primer pairs used in PCR/*q*PCR experiments in this work.

**Table 2:** Primers used in polymerase chain reaction (PCR) experiments and quantitative PCR (*q*PCR).

Primer	Sequence (5´-3´)	Use
lacZintR	gcctcttcgctattacgcca	Checking of plasmids and mutants
OmegaintR	gggccttgatgttacccgaga	
M13puc-F	gttttcccagtcacgac	
M13puc-R	caggaaacagctatgac	
*nodD2*-F	ctaaccaagccggagga	
*nodD2*-R	ccgaagccgtgtacca	
Y4xi-F	taatcagcctggctgaca	
Y4xi-R	aacagaacgagcgcgtaga	
HHnodD1extF	ctttgagcggtttctcgcag	
HHnodD1extR	gagtatcgaagacggctggg	
nolRupst	ttagctacccccaattcttgc	
nolRdwst	gaaaaagccccgcgattgct	
HHsyrMextF	gtacatcacaagccctcgct	
HHsyrMextR	cgcttgaagggaacctgtga	
syrMintEcoRI-F	atcgaattcgcgtgacatgttcaatgacg	Construction of pMUS1492
syrMintXbaI-R	gcttctagagtggatctacaatagcgagc	
syrMPMP92bis-F KpnI	gccggtacctgagggaatggtggagaagg	Construction of pMUS1514
syrMPMP92bis-R XbaI	ctttctagaatcaatcgcacgcggtgtc	
NodD1NKpnIsinstop	aaaggtacccggaggcatgtaggcaattg	
NodD1CKpnIstop	aaaggtacccgttagaggcatgtaggcaa	
NodD1NXbaI	aggtctagagatgcgttttaagggcctt	
NodD2NXbaI	aggtctagagatgcgttttaagggactt	
NodD2NKpnIsinstop	aaaggtacccggggtcgatattccactga	
NodD2CKpnIstop	aaaggtacccgctagggtcgatattccac	
rt16S-F2	gataccctggtagtccac	*q*PCR
rt16S-R2	taaaccacatgctccacc	
qnodA-F^a^	cgtcatgtatccggtgctgca	
qnodA-R^a^	cgttggcggcaggttgaga	
qnodD1-F	gcgagcacggactgcgaa	
qnodD1-R	cgggaaaaatgggttgcgga	
qnodD2-F	acgctaaagccctccatcga	
qnodD2-R	atggtggaagtgccagtgga	
rt-nolR-F	ccaaaacgcctgctcatt	
rt-nolR-R	attctgggcacgcaactt	
qsyrM-F^a^	gttcaatgacgatctcttggt	
qsyrM-R^a^	attgccatagttaccttcgac	
qttsI-F	cggttggaagatcaactcta	
qttsI-R	gtcaattcaagaacgtagcc	
qpsfHH103d_161-F	agaatgtcgcatacctcttag	
qpsfHH103d_161-R	gtgaaggctgttatcccatc	
qpsfHH103d_208-F	gatctcaggctttcacagac	
qpsfHH103d_208-R	cgtcctctgacggtttcatg	
qpsfHH103d_229-F	gctacgcatcaaagtggaag	
qpsfHH103d_229-R	gttggggttctcaaagatgaa	
qpsfHH103d_255-F	aggcggttacattgctac	
qpsfHH103d_255-R	atcccactgcaccacttt	
qpsfHH103d_257-F	acagacagctaaattctctgc	
qpsfHH103d_257-R	gatgttgtcatcctctggata	
qpsfHH103d_275-F	gagcgcaatatcgcatgt	
qpsfHH103d_275-R	ctcaaccaacgacacgaa	
qpsfHH103d_292-F	gtggctttcaatatcggg	
qpsfHH103d_292-R	cggtgtagtttgtccaga	
qpsfHH103d_306-F	cttcacagttacggagga	
qpsfHH103d_306-R	gcgttcgcgagatcaaaa	
qpsfHH103d_327-F	gagctggatcatggcaa	
qpsfHH103d_327-R	atgctgccaatcaagca	
qpsfHH103d_373-F	tcgacgattcaataagggtg	
qpsfHH103d_373-R	catatcctctccgcaatagc	
qpsfHH103d_448-F	actctcaagagcaggattagg	
qpsfHH103d_448-R	accatcgggagtagtatcagt	
qpsfHH103d_3504-F	caaaggggggcatgga	
qpsfHH103d_3504-R	caaccgatcgaagagcta	
qSFHH103_00346-F	tgctgaattcctcggaag	
qSFHH103_00346-R	cagcatcgacttgacgaa	
qSFHH103_04163-F	acgtgggtggaaacga	
qSFHH103_04163-R	gacgaatctgtctcgaca	

**^a^** The gen ID of *nodA* and *syrM* are psfHH103d_126 and psfHH103d_367, respectively, in **Supplementary Data Sheet 5**.

#### 2.1.1 Construction of plasmids

Plasmid pMUS789 is a pK18*mob* (Schäfer et al., 1994) derivative carrying a *nodD2*::*lacZ*Δp-Gm^R^ fusion. For constructing pMUS789, a ~5,7 kb *EcoR*I fragment containing the *nodD2*::*lacZ*Δp-Gm^R^ fusion from pMUS788 (Acosta-Jurado et al., 2019) was subcloned into pK18*mob*.

Plasmid pMUS859 is a pK18*mob* derivative carrying a *nolR*::*lacZ*Δp-Gm^R^ fusion. For generating this plasmid, the *lacZ*Δp-Gm^R^ cassette from pAB2001 was subcloned as a 4.3 kb *Nco*I fragment into the *Nco*I site of pMUS672, a pBluescript derivative carrying HH103 *nolR* (Vinardell et al., 2004b), rendering plasmid pMUS857. Finally, a 7,0 kb *EcoR*I fragment containing the *nolR*::*lacZ*Δp-Gm^R^ fusion from pMUS857 was subcloned into pK18*mob*, generating plasmid pMUS859.

Plasmid pMUS1492 is a pK18*mob* (Schäfer et al., 1994) derivative carrying an internal fragment of the HH103 *syrM* gene. For constructing this plasmid, an internal fragment of *syrM* was PCR amplified by using primers syrMintEcoRI-F and syrMintXbaI-R, and gDNA of HH103 as template. The amplified fragment was digested with *Eco*RI and *Xba*I and subcloned into pK18*mob*, rendering plasmid pMUS1492.

For constructing a broad host-range containing the HH103 *syrM*, a fragment containing this gene and its NB was PCR amplified by using primers SyrMextBamHI-F and SyrMextXbaI-R, and HH103 gDNA as template. The amplified fragment was digested with *Bam*HI and *Xba*I and subcloned into pBBR1MCS-2 (Kovach et al., 1995), rendering plasmid pMUS1514.

#### 2.1.2 Construction of *Sinorhizobium fredii* HH103 single and double mutants

In this work we have used a number of HH103 single or double mutants in the regulatory genes *nodD1*, *nodD2*, *nolR*, *ttsI*, and *syrM* (see **Table 1**). In all the double mutants, one of the genes was mutated by insertion of the *lacZ*Δp-Gm^R^ cassette (Becker et al., 1995), whereas the second gene was either deleted or inactivated by insertion of the Ω interposon (Prentki and Krisch, 1984) or the plasmid pK18*mob* (Schäfer et al., 1994). Although many of the mutants had been already constructed in our laboratory, others have been generated in this work. For that purpose, we have used mutated versions of one of the genes subcloned in pK18*mob*, a Km^R^ vector that is suicide in rhizobia, and transferring the corresponding plasmids from *E. coli* to rhizobia by triparental mating using the helper plasmid pRK2013 (Figurski and Helinski, 1979; Simon, 1984). The specific details of the mutants constructed in this work are provided in **Supplementary Table S1**. All these mutants were checked by hybridization and by PCR.

### 2.2 Transcriptomic analyses

*Culture conditions and RNA extraction.* Three strains of *Sinorhizobium fredii*, SVQ269 (= HH103 Rif^R^), SVQ515 (= HH103 Rif^R^ *nodD2*::Ω) and SVQ548 (= HH103 Rif^R^ *nolR*::*lacZ*-Gm^R^), were cultivated at 28°C until stationary phase (OD_600_ ≈ 1,2) on yeast extract mannitol medium (YM) supplemented with the appropriate antibiotics. Genistein at a concentration of 3.7 μM was added to the medium when required. Two independent total RNA extractions, carried out as describe above for *q*PCR experiments, were performed out for each condition.

*RNA-seq experiments.* RNA-seq experiments were conducted as previously described (Pérez-Montaño et al., 2016; Acosta-Jurado et al., 2019; Acosta-Jurado et al., 2020). Depletion of rRNA was performed with the MICROB Express Bacterial mRNA Purification kit (Ambion). RNA quality assays and sequencing was performed by Sistemas Genómicos (https://www.sistemasgenomicos.com/web_sg) by using an Illumina HiSeq 2000 sequencing instrument (Illumina). High-quality were mapped against HH103 genome (http://www.ncbi.nlm.nih.gov/assembly/GCF_000283895.1/).

*Gene Prediction and Expression Analysis.* Gene expression levels were quantified as previously described (Anders and Huber, 2010; Robinson et al., 2010; Trapnell et al., 2010; Anders et al., 2015). More details can be found in Pérez-Montaño et al. (2016) and Acosta-Jurado et al. (2019); Acosta-Jurado et al. (2020). We considered as differentially expressed genes (DEGs) those showing a fold-change lower than -3 or higher than +3 compared to the value previously reported for HH103 grown in the absence of genistein, being the p-value lower than 0.05 (Pérez-Montaño et al., 2016).

*RNA-seq data accession number.* All the RNA-seq data used in this publication can be consulted in the Sequence Read Archive of NCBI (BioProject database, BioProject IDs PRJNA313151 and PRJNA413684.

### 2.3 Plant tests

Nodulation tests on *Lotus japonicus* ecotype Gifu were performed as described by Acosta-Jurado et al. (2016a). At least two independent assays were carried out for each strain, giving similar results. The figures included in this paper shows a representative experiment. In all the assays, *Mesorhizobium loti* MAFF 303099 was used as positive control of effective nodulation in *L. japonicus*. In each experiment, we used five Leonard jar assemblies containing four germinated seeds each per treatment (n = 20). The upper vessel contained 220 ml of sterilized vermiculite supplemented with the plant nutrient solution described by Rigaud and Puppo (1975), whereas the lower recipient was filled with 180 ml (pH 7.0) of the same solution. Each Leonard jar was inoculated with 1 mL of a medium exponential phase rhizobial culture (OD_600_ ≈ 0,5-0,6), containing approximately 10^8^ bacteria. Upon inoculation, plants were grown for 9 weeks in a plant-growth chamber (16 h photoperiod, 25 °C in the light and 18 °C in the dark). Nitrogenase activity in nodules was estimated by acetylene reduction assays (ARA) as described by Buendía-Clavería et al., 1986.

### 2.4 Identification of Nod factors

Purification and LC-MS/MS determination of NFs produced by *S. fredii* strains were carried out as described by Acosta-Jurado et al. (2019). Two independent cultures in B- medium were used for each strain and condition (presence or absence of genistein). In previous works we showed that HPLC-HRMS signal areas reflect the relative abundance of each NF, allowing the estimation of quantitative variations for any individual NF. Samples used in this work were analysed the same day to minimize variations due to experimental conditions.

## 3 Results

### 3.1 Inactivation of either *nolR* or *nodD2* of *Sinorhizobium fredii* HH103 affects the expression of hundreds of genes in the presence of genistein

In previous works we investigated the effect of genistein in the transcriptomic profiles of HH103 and mutant derivatives affected in the symbiotic regulatory genes *nodD1*, *ttsI*, and *syrM* (Pérez-Montaño et al., 2016; Acosta-Jurado et al., 2020). These RNA-seq studies allowed us to define a set of 100 HH103 differentially expressed genes (DEGs) in the presence of that flavonoid, the so-called *nod* regulon (Pérez-Montaño et al., 2016). In order to better understand the effect of genistein in the expression of the *nod* regulon of HH103, in this work we have used a transcriptomic approach (RNA-seq) to study the effect of genistein in two other mutant derivatives in HH103 symbiotic regulatory genes: *nodD2* (strain SVQ515) and *nolR* (strain SVQ548). These analyses have been performed both in the absence and presence of genistein, and the data obtained have been compared to the results obtained for the wild-type strain. As we have done in previous transcriptomic studies, we considered as DEGs those genes that, in the presence of genistein, exhibited a fold-change in their expression lower than -3 (i. e., <0.33) or higher than +3 in comparison to the values obtained for the wild-type strain grown in the absence of genistein (**Supplementary Data Sheets 1 and 2**, respectively). The numbers of DEGs found in each mutant, 382 in HH103 *nodD2* and 201 in HH103 *nolR*, were higher than that found in the wild-type strain, 100 (Pérez-Montaño et al., 2016). These results indicate that both regulatory genes have a high impact in HH103 gene expression. The number of common DEGs found in these mutants was 122 (83 of them belonging to the *nod* regulon), whereas 79 and 260 genes were found as DEGs only in the *nolR* and in the *nodD2* mutant backgrounds, respectively (**Supplementary Data Sheet 3**).

**Figure 1:**
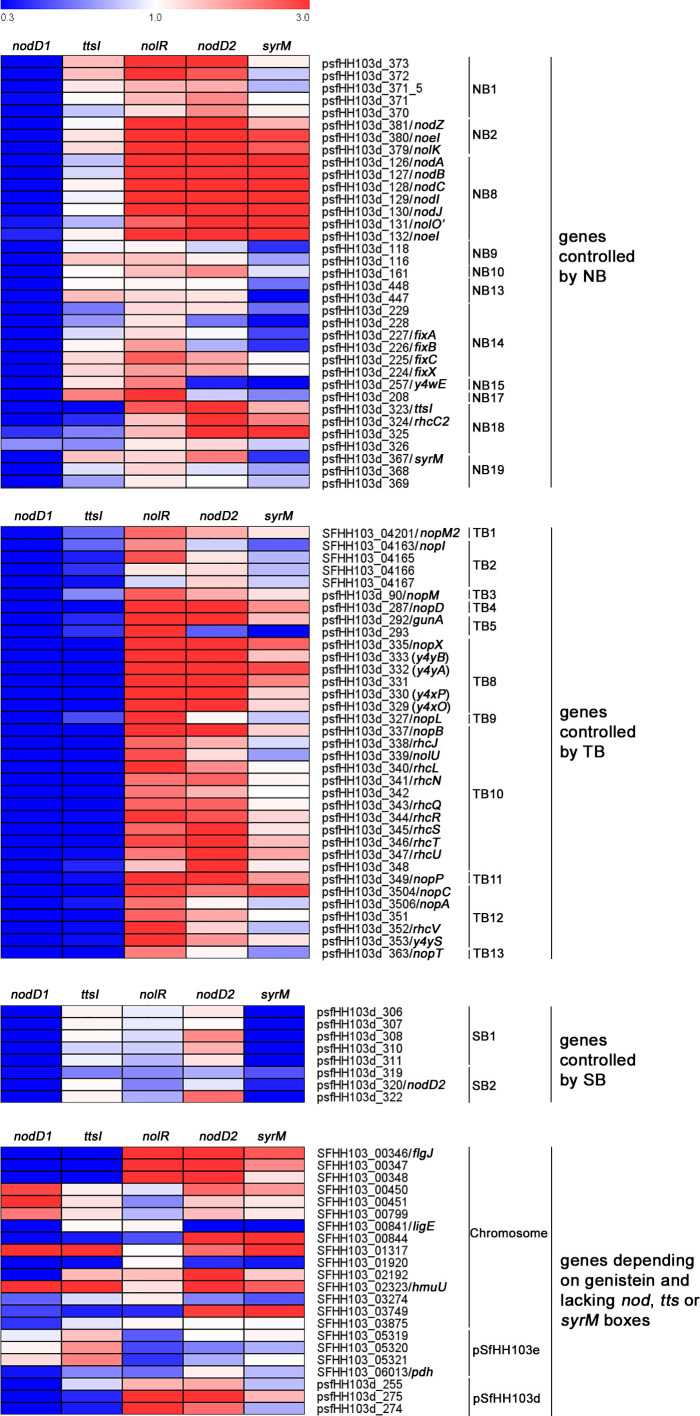
Heat map of the effect of the inactivation of either *nodD1*, *nodD2*, *nolR*, *syrM*, or *ttsI* on the expression of the HH103 *nod* regulon upon treatment with genistein when compared to the expression observed in the wild-type strain grown in the presence of this flavonoid.

The 100 DEGs found in the wild-type strain upon induction with genistein can be divided into different groups according to the presence of known motifs in their promoter sequences, such as NBs, TBs, or SyrM boxes (Pérez-Montaño et al., 2016; Acosta-Jurado et al., 2020) As mentioned above, 83 out of these 100 genes were also found as DEGs in both the *nolR* and *nodD2* mutants in the presence of genistein when compared to the wild-type strain grown in the absence of genistein, whereas 11 and 3 genes were found only in the *nolR* or in the *nodD2* mutant respectively. All these 97 genes are marked in bold letters in **Supplementary Data Sheet 3**. **Supplementary Data Sheet 4** shows the expression level of the 100 DEGs found in HH103 upon treatment with genistein in the HH103 *nodD1*, *nodD2*, *nolR*, *syrM*, and *ttsI* mutants grown in the presence of this flavonoid, as well as the comparison to the values obtained in the wild-type strain. The expression levels for the *nodD1*, *ttsI*, and *syrM* mutants have been previously published by our group (Pérez-Montaño et al., 2016; Acosta-Jurado et al., 2020). We considered as overexpressed or repressed in the different mutants analysed those genes whose expression in the presence of genistein was more than three times higher or lower in the mutant than in the wild-type strain grown in the presence of genistein. The presence of well conserved NolR-binding boxes in the upstream region of these genes or operons (Acosta-Jurado et al., 2019) is also indicated in **Supplementary Data Sheet 4**. For validating RNA-seq data, we studied the expression of 16 out of these 100 genes by *q*PCR in the *nolR* and *nodD2* mutants. **Supplementary Data Sheet 5** shows the fold-change values of these 16 genes (by both *q*PCR and RNA-seq) in both mutant strains in the presence of genistein with respect to their expression in the wild-type strain supplemented with genistein. In most cases, there is a good correlation between the two types of data. A heat map showing the effect of the lack of either NodD1, NodD2, NolR, SyrM, or TtsI on the expression of the 100 genes belonging to the HH103 *nod* regulon is provided in **Figure 1**.

Regarding genes depending on well-defined NBs, all HH103 genes that have been found to be related to Nod factor production (both common and specific) were overexpressed in the presence of genistein in the *nodD2* and *nolR* mutants, similarly to that previously observed for the *syrM* mutant (Acosta-Jurado et al., 2020): the *nodZnoeInolK* (NB2, involved in the fucosylation of NFs; Lamrabet et al., 1999) and *nodABCIJnolO’noeI* (NB8, responsible of the synthesis of the Nod factor backbone and the methylation of the fucosyl residue; Madinabeitia et al., 2002). Also in both mutants, the psfHH103d_373 gene (NB1) shows an expression level that is more than 3-fold higher when compared to the wild-type strain. This gene codes for a hypothetical protein that remains uncharacterized (Vinardell et al., 2015). In the *nolR* mutant, but not in the *nodD2* mutant, gene psfHH103d_208 (NB17) is also overexpressed. To our knowledge, the encoded product of this gene, the putative periplasmic component of an ABC-type transport system (Vinardell et al., 2015), has not been studied in rhizobia so far. In coherence with the expression data, a well conserved NolR-binding box is present in the upstream region of all these genes/operons (**Supplementary Data Sheet 4**, Acosta-Jurado et al., 2019). On the other hand, psfHH103d_323 (NB18), which codes for TtsI (López-Baena et al., 2008), is clearly upregulated in the *nodD2* mutant (5.1 when compared to the wild-type) but only slightly in the *nolR* derivative (2.6-fold). Interestingly, psfHH103d_257 (NB15), which codes for an enzyme that participates in indole-3-acetic acid synthesis (Vinardell et al., 2015) is slightly overexpressed (2.3-fold) and repressed (0.4-fold) in the *nolR* and *nodD2* mutants, respectively. For both psfHH103d_323 and psfHH103d_257 a NolR-binding box has been located upstream of these genes.

Concerning genes depending on TBs, most of them were upregulated, some of them slightly, in both mutant backgrounds (**Supplementary Data Sheet 4**). These results reveal that, as expected, inactivation of either *nolR* or *nodD2* increases the expression of the HH103 symbiotic T3SS since both NolR and NodD2 repress *ttsI* expression. For genes under the control of TB2, 4, 5, 8, 9, 11, and 12, the presence of NolR-binding boxes in their promoter regions might also contribute to the observed increase in their expression in the *nolR* mutant background (**Supplementary Data Sheet 4**, Acosta-Jurado et al., 2019).

In *S. fredii*, HH103 only two groups of genes present a well-defined SyrM box in their upstream DNA sequences and, accordingly, its expression depends on the presence of SyrM (**Supplementary Data Sheet 4**, Acosta-Jurado et al., 2020). Inactivation of *nolR* had no effect on the expression of these genes, but that of *nodD2* slightly increased the expression of some of them.

Regarding genes lacking NBs, TBs, or SyrM boxes in their promoter sequences, both the chromosomal SFHH103_00346-SFHH103_00348 and the symbiotic plasmid psfHH103d_275-psfHH103d_274 genetic regions were upregulated in both the *nodD2* and *nolR* mutant backgrounds. SFHH103_00346, SFHH103_00347 and SFHH103_00348 codes for flagellar proteins (FlgJ, FlgN, and MotF), as described recently in *S. meliloti* (Sobe et al., 2022). The predicted products coded by psfHH103d_275 and psfHH103d_274 are hypothetical proteins. Three other genes were overexpressed in the *nodD2* but not in the *nolR* mutant: SFHH103_00844 (conserved hypothetical protein containing nucleic acid-binding domains), SFHH103_02192 (peptidase_M10_C and COG2931 domain-containing conserved hypothetical protein) and SFHH103_02323/*hmuU* (a putative iron ABC transporter permease). In addition, the SFHH103_00841/*ligE* (glutathione S-transferase etherase domain-containing protein) was clearly repressed in the *nodD2* mutant.

### 3.2 *Lotus japonicus* root exudates have a bigger impact in the transcriptomic profile of the HH103 *nodD2* and *nolR* mutants than genistein

In a previous work we reported the effect of *L. japonicus* root exudates on the global gene expression of HH103 and its *nodD2* and *nolR* mutant derivatives (Acosta-Jurado et al., 2019). In this work we have carried out these transcriptomic analyses upon treatment with genistein. The number of DEGs found in the presence of root exudates were higher than that found in the presence of genistein: 575 vs 382 and 279 vs 201 for HH103 *nodD2* and HH103 *nolR* respectively. **Supplementary Data Sheet 6** summarizes the transcriptome data for both mutants in both conditions (genistein or *L. japonicus* root exudates).

**Figure 2:**
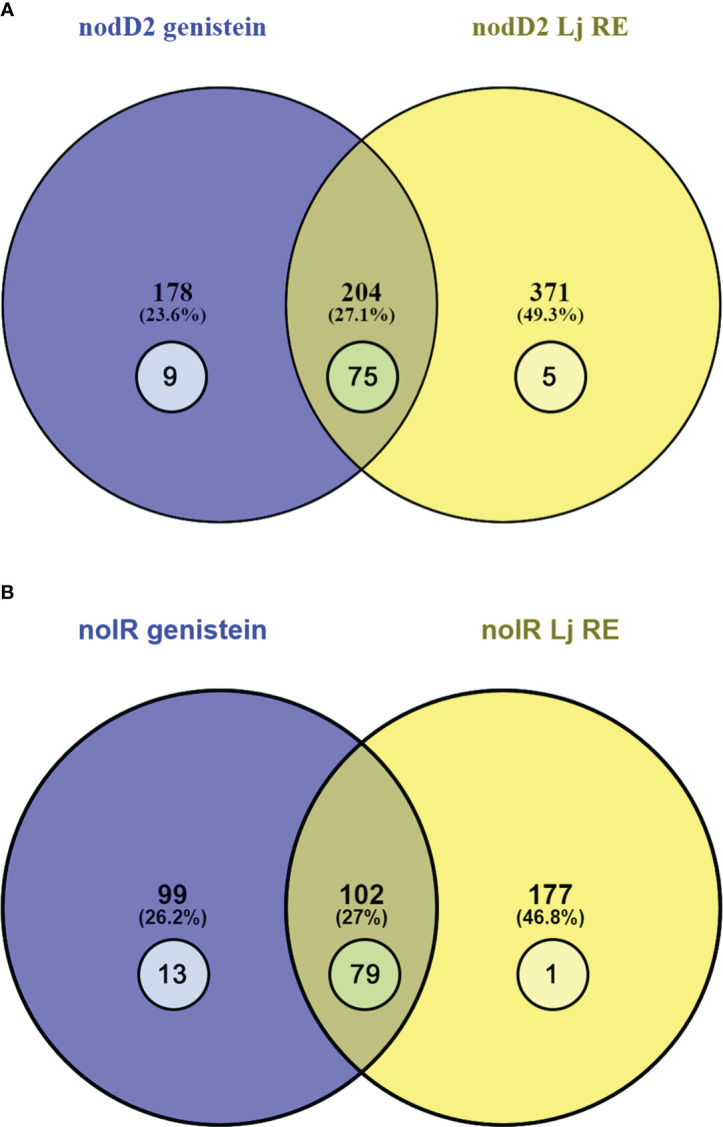
Venn diagram of the sets of the DEGs found in the HH103 *nodD2* **(A)** and *nolR* **(B)** mutants upon treatments with genistein (blue circle) and *L. japonicus* root exudates (yellow circle). The numbers represented inside circles correspond to genes belonging to the *nod* regulon.

In the case of the HH103 *nodD2* mutant, 371 and 178 DEGs were exclusively found upon treatment with *L. japonicus* root exudates and genistein respectively, whereas 204 DEGs were found in both conditions, 77 belonging to the *nod* regulon (**Supplementary Data Sheet 7**, **Figure 2A**). In the case of HH103 *nolR*, 177 and 99 DEGs were detected exclusively with root exudates and genistein, respectively, and the number of shared DEGs were 102, 79 belonging to the *nod* regulon (**Supplementary Data Sheet 7**, **Figure 2B**). For both mutants, most genes involved in NF production or T3SS functioning were affected by treatments with either genistein or *L. japonicus* root exudates.

### 3.3 Analysis of the expression of different HH103 symbiotic regulators (*nodD1*, *nodD2*, *nolR*, *syrM*, *ttsI*) by RNA-seq and *q*PCR

One of the main purposes of this work was to study the possible connections among different symbiotic regulators previously identified in HH103: *nodD1*, *nodD2*, *nolR*, *syrM*, and *ttsI*. **Table 3** summarizes the fold-changes, as determined by RNA-seq, of the expression of these genes in the wild-type strain and in different individual mutants in the mentioned genes when grown in the presence of genistein, in comparison to the values obtained in the wild-type strain grown in the absence of genistein. The *nodA* gene, coding for one of the key enzymes participating in NF biosynthesis, was included as a control of a gene whose expression is affected by different symbiotic regulators. In addition, we performed *q*PCR analyses of the expression of all these six genes (*nodA* and the five symbiotic regulators previously mentioned) in the same genetic backgrounds as those employed in the RNA-seq analyses (**Table 3**).

**Table 3:** RNA-seq and *q*PCR analyses of the expression of *nodA* and several genes coding for symbiotic regulators of *S. fredii* HH103 when grown in genistein.

		Fold change in different genetic backgrounds in the presence of genistein^1^					
		Wild-type	*nodD1*	*ttsI*	*nodD2*	*nolR*	*syrM*
Gen ID	Gene name	RNA-seq *^2^*					
psfHH103d_126	*nodA*	17.65	0.72	14.68	233.98	102.56	77.22
psfHH103d_386	*nodD1*	0.80		0.76	1.36	0.96	0.85
psfHH103d_323	*ttsI*	10.09	0.82		51.79	26.68	17.76
psfHH103d_320	*nodD2*	6.60	0.92	6.95		4.38	2.56
SFHH103_02239	*nolR*	0.99	0.93	1.20	0.54		0.32
psfHH103d_367	*syrM*	5.81	0.86	9.29	13.36	8.23	
Gen ID	Gene name	*q*PCR ^3^					
psfHH103d_126	*nodA*	31.71	1.19	31.56	225.45	244.16	201.55
psfHH103d_386	*nodD1*	0.84		0.83	1.96	1.16	0.95
psfHH103d_323	*ttsI*	13.27	1.32		98.36	61.53	28.84
psfHH103d_320	*nodD2*	6.04	0.70	4.48		6.21	2.73
SFHH103_02239	*nolR*	1.18	1.24	1.45	1.36		0.79
psfHH103d_367	*syrM*	6.51	0.85	9.80	9.29	13.02	

^1^With regard to the expression in the wild-type strain in the absence of genistein.

^2^p values were lower than 10^-3^.

^3^Standard deviations were less or equal than 15% of the average fold-change.

As expected, both methodologies showed that *nodA* expression was strongly enhanced by genistein in a NodD1-dependent manner. The absence of either NodD2, NolR, or SyrM provoked strong increases of this expression, whereas lack of TtsI had no effects at all. Similarly to *nodA*, both *ttsI* and *syrM* are preceded by a NB (Vinardell et al., 2015). As shown in **Table 3**, the expression of these two regulatory genes is, as that of *nodA*, genistein-induced in a NodD1 dependent manner, and repressed (in a lesser extent in the case of *syrM*) by NolR and NodD2. In addition, both RNA-seq and *q*PCR data showed that SyrM and TtsI appear to repress the expression of each other. Regarding *nodD1*, its expression was slightly repressed by the presence of genistein both in the wild-type and in the *ttsI* mutant background, and slightly increased in the absence of NodD2. The *nodD2* gene also showed genistein-induction and dependence on the presence of functional copies of NodD1 and, to a lesser extent, SyrM. Regarding *nolR*, its expression was not affected by genistein nor by NodD1, NodD2, or TtsI. However, the absence of SyrM appeared to negatively affect the level of transcripts of *nolR*, as scored by both RNA-seq and *q*PCR.

### 3.4 Assessing the fine-tuning modulation of the expression of *nodD1*, *nodD2*, *nolR*, *syrM* and *ttsI* by β-galactosidase assays

**Figure 3:**
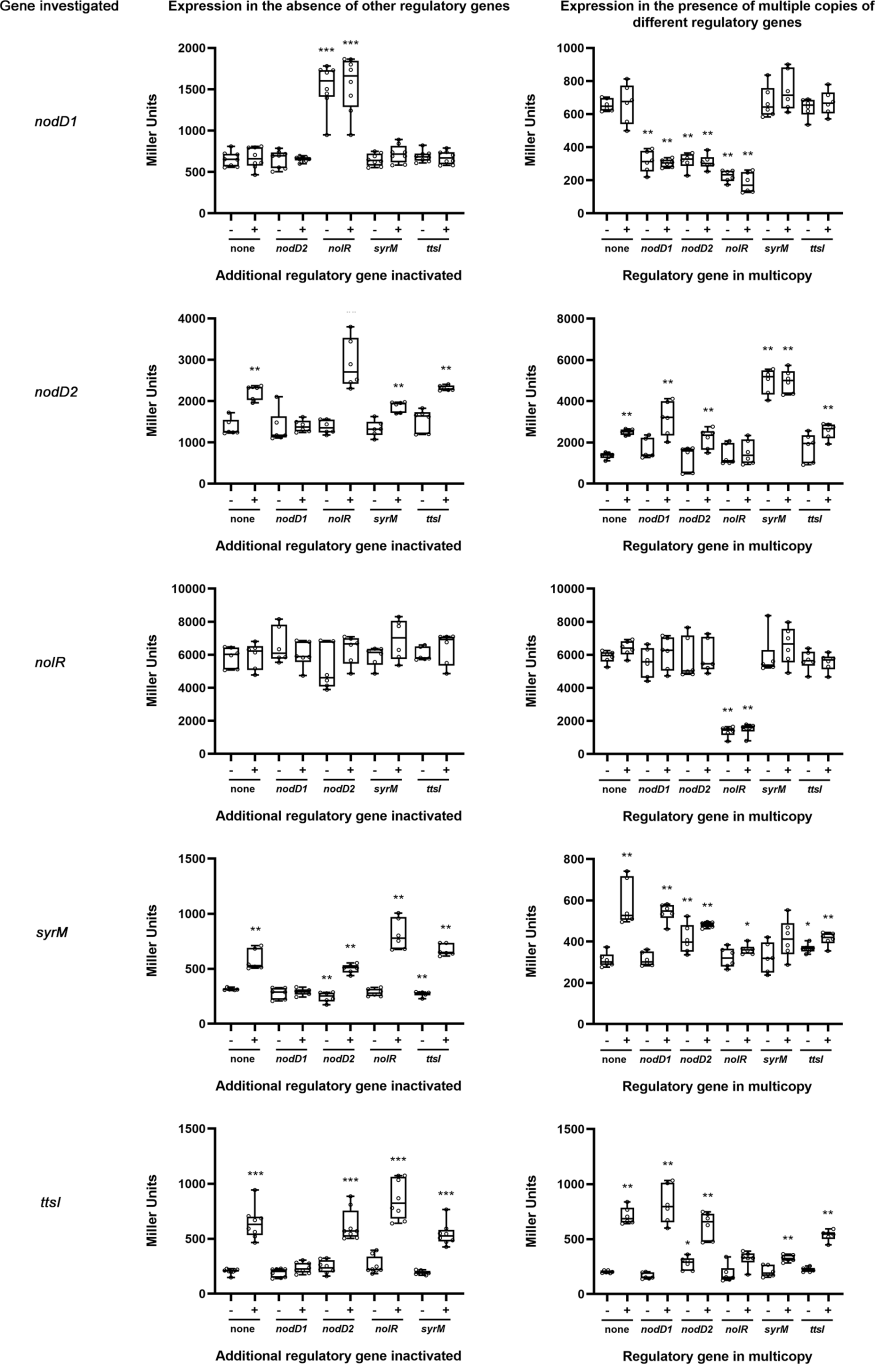
Analysis of *nodD1*, *nodD2*, *nolR*, *syrM*, and *ttsI* expression by β-galactosidase assays in the absence of an additional regulatory gene (left panels) or in the presence of multiple copies of different regulatory genes (right panels). These assays were carried out in the absence (-) or presence (+) of genistein 3.7 µM. Error bars show standard deviations. All the treatments were individually compared to the control values (none) obtained in the absence of flavonoids by using the non-parametric test of Mann-Whitney (p<0.001, ***; p<0.002, **, p<0.05, *). Other statistical analyses are indicated in the text.

To further investigate the relationships among different symbiotic regulators (*nodD1*, *nodD2*, *nolR*, *syrM* and *ttsI*) of HH103, we performed β-galactosidase assays of HH103 mutants in each of these genes constructed by insertion of the *lacZ*Δp-Gm^R^ cassette (Becker et al., 1995). In these mutants, the cassette not only disrupts the gene of interest but also provides a readout for expression from the promoter activity of the mutated gene. We carried out two different series of experiments (**Figure 3**): (i) HH103 double mutants in two regulatory genes, one of them carrying the *lacZ*Δp-Gm^R^ cassette, and the other mutated by in frame deletion or by insertion of the Ω cassette (Prentki and Krisch, 1984), in order to analyse the effect of the lack of the latter gene on the expression of the former; (ii) HH103 mutants in a symbiotic regulatory gene with the *lacZ*Δp-Gm^R^ cassette and harbouring extra copies of each of the different symbiotic regulators carried on a plasmid stable in rhizobia, in order to analyse the effect of multiple copies of the latter gene on the expression of the former one. The differences were analysed by using the non-parametric test of Mann-Whitney. For the sake of clarity, only the statistical differences between each treatment and the control (the corresponding *lacZ*Δp-Gm^R^ mutant grown in the absence of genistein) are shown in **Figure 3**. When necessary, additional statistical comparisons are mentioned below.

Concerning *nodD1*, its expression was not affected by the absence of either *nodD2*, *ttsI*, or *syrM*, but it significantly increased (2.3-fold) in the absence of a functional copy of *nolR*. On the other hand, *nodD1* expression was significantly repressed in the presence of extra copies of either *nodD2*, *nolR* and, also, *nodD1* (2.1, 3.2, and 2.1-fold respectively), but not when multiple copies of either *ttsI* or *syrM* were present. All the effects on the expression of *nodD1* mentioned were found regardless of the absence or presence of genistein.

The expression of *nodD2* was slightly, but significantly, increased (1.6-fold) in the presence of genistein, but this induction did not take place in the absence of NodD1. The genistein-induction of *nodD2* was slightly, but significantly, higher (2.1-fold, p<0.05) and lower 1.4-fold, p<0.002)) in a *nolR* and a *syrM* mutant backgrounds, respectively, and not affected by the absence of TtsI. Upon treatment with genistein, the presence of extra copies of *nodD2* and *nodD1* slightly affected *nodD2* expression in a negative and positive way, respectively, but these expression changes were not significant. However, when multiple copies of *syrM* were present, the expression of *nodD2* enhanced significantly in the presence of genistein (2-fold, p<0.002) and was independent of the presence of genistein. On the other hand, the presence of extra copies of NolR negated the genistein-dependent induction of *nodD2* expression, provoking a significant difference with respect to the control strain (p<0.002).

The absence of either *nodD1*, *nodD2*, *syrM*, or *ttsI* did not significantly affect *nolR* expression, whereas the presence of extra copies of this gene, but not that of either *nodD1*, *nodD2*, *syrM*, or *ttsI*, clearly repressed its expression (4.3-fold).

The analysis of the β-galactosidase data in the *syrM*::*lacZ*Δp-Gm^R^ mutant revealed that this gene is subjected to a complex regulation. On the one hand, the expression of *syrM* almost duplicated in the presence of genistein (1.84-fold) and it was dependent on the presence of NodD1. Also, when genistein was present, the absence of NolR provoked a slight but significant increase of *syrM* expression (p<0.05). On the other hand, the presence of extra copies of either *nodD2*, *nolR*, *syrM*, or *ttsI* exerted a significant repressor effect on *syrM* expression upon treatment with genistein (p<0.002 for extra copies of *nodD2*, *nolR*, and *ttsI*; p<0.05 for extra copies of *syrM*).

As expected, the expression of *ttsI* was enhanced in the presence of genistein (more than 3-fold) in a NodD1-dependent manner. This genistein-dependent expression was not affected by the absence of NodD2 or SyrM, but exhibited a slight but significant increase when NolR was not present (p<0.05). On the other hand, the presence of extra copies of *nolR*, *syrM*, or, to a lesser extent, *ttsI*, significantly reduced the expression of this gene upon treatment with genistein (p<0.002 in all cases).

### 3.5 Phenotype of HH103 symbiotic regulatory gene double mutants on *Lotus japonicus*

**Figure 4:**
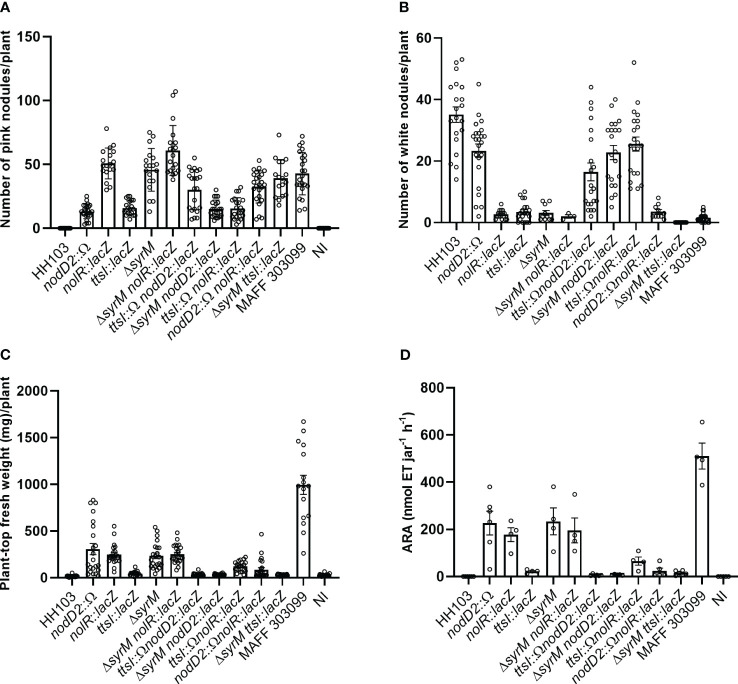
Symbiotic performance of *S. fredii* HH103 and different mutant derivatives with *L. japonicus*. **(A)** Number of pink nodules per plant. **(B)** Number of white nodules per plant. **(C)** Plant-top fresh weight per plant. **(D)** ARA per Leonard jar. Error bars show standard deviations. For each HH103 individual mutant in either *nodD2*, *nolR*, *syrM*, or *ttsI*, the values of these parameters were compared to those of their corresponding double mutants, the wild-type strain and the Non-inoculated plants (NI) by using the non-parametric test of Kruskal-Wallis.

HH103 is a broad-host range rhizobial strain able to induce the formation of Fix^+^ nodules in *Lotus burttii* but ineffective white empty nodules in *L. japonicus* (Acosta-Jurado et al., 2016a). However, HH103 mutants in the secondary symbiotic regulators *nodD2*, *nolR*, *syrM*, or *ttsI* gain the ability to fix nitrogen with *L. japonicus*, although they differ in their effectiveness (Acosta-Jurado et al., 2019; Acosta-Jurado et al., 2020; Jiménez-Guerrero et al., 2020). In order to shed light about this issue, in this work we have studied the symbiotic behaviour with *L. japonicus* of all the possible combinations of double mutants in the four genes mentioned above. Four different parameters were analysed (**Figure 4**): plant-top fresh weight (PTFW), number of white nodules, number of pink nodules, and nitrogenase activity (estimated by acetylene reduction assay, ARA). **Supplementary Figure S1** provides individual analysis for strains sharing a mutation on each specific regulatory gene.

As previously observed, HH103 individual mutants in either *nodD2*, *nolR*, or *syrM* showed similar symbiotic performances (values of PTFW and ARA), which were higher than that of HH103 *ttsI*. In contrast to HH103, all the double mutants tested in this work were able to induce the formation of nitrogen-fixing nodules in *L. japonicus*, as well as white nodules (**Figure 4**), as HH103 did. However, the number of both types of nodules were highly variable among the different strains tested.

Regarding PTFW (**Figure 4**), the three double mutants lacking TtsI behaved similarly to the individual HH103 *ttsI* mutant. These results indicate that carrying a mutation in *ttsI* led to a decrease in the symbiotic performance of the HH103 mutants in either *nodD2*, *nolR*, or *syrM*. A similar pattern could be observed in the case of ARA (**Figure 4**). However, it is noticeable that the mutation of *nolR*, but not those of *nodD2* or *syrM*, increased the symbiotic performance (PTFW and ARA) of the HH103 *ttsI* mutant (**Figure 4**, **Supplementary Figure S1**).

Regarding the different combinations of double mutants of the *nodD2*, *nolR*, and *syrM* genes, only HH103 *nolR syrM* exhibited a symbiotic performance similar to that of the corresponding individual mutants. Instead, plants inoculated with either HH103 *nodD2 nolR* or HH103 *nodD2 syrM* exhibited values of PTFW and ARA that were clearly smaller than those exhibited by the corresponding individual mutants.

### 3.6 Production of Nod factors is *Sinorhizobium fredii* HH103 double mutants in the *nodD2*, *nolR* and, *syrM* genes

In previous works (Acosta-Jurado et al., 2019; Acosta-Jurado et al., 2020) we showed that the HH103 individual mutants in the *nolR*, *nodD2*, and *syrM* genes exhibit increased expression of genes involved in NF synthesis and, consequently, enhanced production of these signal molecules. In this work we have investigated the production of NFs in the different combinations of HH103 double mutants in the *nolR*, *nodD2*, and *syrM* genes and compared to that of the wild-type strain and the single mutants in these regulatory genes.

HPLC coupled with data-dependent High-Resolution Mass Spectrometry (HPLC-HRMS/MS) enabled the identification of 108 different NFs in HH103 cultures upon induction with genistein (as detailed in **Supplementary Data Sheet 8**). Interestingly, the number of different NFs was notably higher in double (129, 128 and 118 in *nolR nodD2*, *syrM nodD2*, and *syrM nolR*, respectively) and, particularly, in single mutants (158, 145 and 170 in *nodD2*, *nolR* and *syrM*, respectively) than in the wild-type strain (**Table 4**, **Supplementary Data Sheet 8**). All these mutants, single or double, share a common core of 65 different NFs with the parental strain (**Supplementary Data Sheet 8**). The signal area values registered by the mass spectrometer for a particular NF serves as a metric for comparing the production of these molecules among the tested strains subjected to identical treatment conditions, as previously shown by Acosta-Jurado et al. (2019); Acosta-Jurado et al. (2020) and Jiménez-Guerrero et al. (2020). In this context, most of the common NFs were detected at elevated concentrations in the *nodD2* (58), *nolR* (38), and *syrM* (44) single mutants when compared to those produced by the parental strain (**Table 4**, **Supplementary Data Sheet 8**). Interestingly, a different landscape emerges when analysing the relative amounts of NFs in the double mutants compared to *S. fredii* HH103. While certain shared NFs were also identified at elevated concentrations (31 for *nolR nodD2*, 16 for *syrM nodD2*, or 19 for *syrM nolR*) when compared to those produced by HH103, the majority of them were observed at similar or even lower levels than in the parental strain (36, 49, and 46 for *nolR nodD2*, *syrM nodD2*, and *syrM nolR*, respectively) (**Table 4**, **Supplementary Data Sheet 8**). Overall, these results indicate that a general overproduction of NFs in the presence of genistein is a characteristic shared by all single mutants but not conserved among double mutant strains. In **Table 5**, we have summarized the chemical structures of the NFs that were shared among all single and double mutants but were not produced by the parental strain, as well as those that were overproduced by all single and double mutants with respect to HH103.

**Table 4:** Number and average fold-change values of HPLC-HRMS signal areas for overproduced and decreased Nod Factors produced by single and double mutants of *Sinorhizobium fredii* HH103 in comparison to the parental strain.

HH103	*nodD2*	*nolR*	*syrM*	*nolR nodD2*	*syrM nodD2*	*syrM nolR*
Total number of detected NFs						
108	158	145	170	128	119	118
Number of common NFs						
Overproduced	56	35	38	31	16	19
Neutral	9	30	27	31	24	25
Decreased	0	0	0	3	25	21
Fold-change average of common NFs						
Overproduced	+19,1	+9,5	+6,5	+12,5	+11,0	+10,1
Decreased	–	–	–	-3,0	-9,0	-7,8

**Table 5:** Chemical structures of Nod Factors categorized based on their presence and overproduction in the *nodD2*, *nolR*, and *syrM* single and double mutants of *Sinorhizobium fredii* HH103 in comparison to the parental strain.

NFs shared by all single and double mutants but not produced by HH103
III (C14:0, Ac, MeFuc), III (C14:1, Ac, MeFuc), III (C16:0, Ac, MeFuc), III (C16:1, Ac, MeFuc), III (C16:2-OH, MeFuc), III (C18:1-OH, MeFuc), III (C18:2-OH, MeFuc), IV (C14:0, Ac, MeFuc), IV (C16:1-OH, MeFuc)
NFs overproduced by all single and double mutants
II-Hex-GlcNAc-GlcNAc (C16:0, MeFuc), II-Hex-GlcNAc-GlcNAc (C18:1, MeFuc), III (C14:0, MeFuc), III (C16:0), III (C16:0, MeFuc), III (C16:0, NMe, MeFuc), III (C18:1, NMe, MeFuc), IV (C14:0, MeFuc
NFs overproduced only by all single mutants
III (C18:1, MeFuc), III (C16:0, Fuc), IV (C16:0, NMe, MeFuc), IV (C16:1), IV (C16:1, Ac, MeFuc), IV (C16:1, Fuc), IV (C16:1, MeFuc), IV (C16:1, NMe, MeFuc), IV (C18:2, MeFuc), IV (C18:1-OH, MeFuc), IV (C18:2-OH, MeFuc), V (C16:0, Fuc), V (C16:0, MeFuc)

## 4 Discussion

The rhizobia-legume symbiosis relies on a complex molecular dialogue between two symbionts (López-Baena et al., 2016; Roy et al., 2020; Ghantasala and Choudhury, 2022). The presence of appropriate signals from the plant (flavonoids) affects the expression of many bacterial genes involved in the establishment and progression of the symbiotic interaction (revised by Jiménez-Guerrero et al., 2018). The regulation of the expression of these bacterial symbiotic genes is complex and involves the participation of different transcriptional regulators (Barnett and Long, 2015; López-Baena et al., 2016). In HH103, a broad-host range strain, different transcriptomic studies (Pérez-Montaño et al., 2016; Acosta-Jurado et al., 2020) have revealed that one hundred genes, collectively known as the *nod* regulon, respond to the presence of genistein, an effective flavonoid for HH103 (Vinardell et al., 2004a). Although the LysR family regulator NodD1 acts as the main activator of the HH103 *nod* regulon, other transcriptional regulators, such as TtsI and SyrM, also participate in the genistein effect on the *nod* regulon. Additional transcriptomics studies carried out upon treatment with *L. japonicus* root exudates showed that two others transcriptional regulators, NodD2 and NolR, are also involved in the modulation of the expression of the HH103 *nod* regulon (Acosta-Jurado et al., 2019). In the present work, we show that these two regulatory proteins are also involved in the fine-tuning modulation of this regulon upon treatment with genistein. The number of DEGs found in the HH103 *nodD2* or *nolR* mutants are higher upon treatment with *L. japonicus* root exudates than upon treatment with genistein, suggesting that these two regulatory proteins are global regulators that, in addition to flavonoids, respond to other components that might be present in legume root exudates, such as phenolic acids, organic acids, fatty acids, galactosides, or aminoacids (Pantigoso et al., 2022; Shimamura et al., 2022). In any case, in both mutants a high number of genes belonging to the *nod* regulon are differentially expressed in both conditions (treatments with either genistein or root exudates from *L. japonicus*).

Regarding the effects of the different regulatory proteins analysed in the HH103 *nod* regulon (**Figure 1**, **Supplementary Data Sheet 4**), NodD1 affects positively the expression of 91 out of these 100 genes, including all the genes depending on NBs, TBs, and SyrM boxes, whereas TtsI induces the expression of the 35 genes depending on TBs (Pérez-Montaño et al., 2016; **Supplementary Data Sheet 4**). SyrM is required for the expression of the two operons harbouring a well-defined SyrM *box* in their promoter sequences but can activate or repress of a number of other genes belonging to the *nod* regulon (Acosta-Jurado et al., 2020; **Supplementary Data Sheet 4**). In this work we show that NodD2 have a similar behaviour that SyrM, although its predominant effect is repressor (**Supplementary Data Sheet 4**). NolR, instead, appears as the main repressor of the *nod* regulon, decreasing the expression of 55 different genes. NodD2, NolR, and SyrM share the repressor activity over the two operons (*nodABCIJnolO*´*noeI* and *nodZnoeLnolK*) that are involved in HH103 NFs biosynthesis and export, which may explain the increased NF production ability of the HH103 individual mutants affected in the coding genes of these transcriptional regulators (**Tables 4**, **5**; **Supplementary Data Sheet 8**).

**Figure 5:**
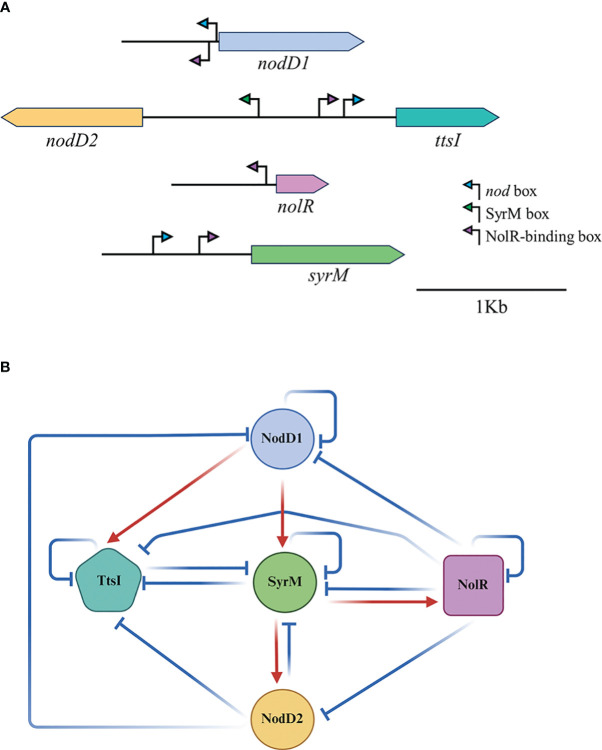
Regulatory relationships between NodD1, NodD2, NolR, SyrM and TtsI in HH103. **(A)** Regulatory motifs present in the upstream regions of the coding genes of these regulators. **(B)** A schematic model indicating the relations of induction (red arrows) or repression (blue lines) in the presence of genistein among the different HH103 symbiotic regulators analysed in this work (NodD1, NodD2, NolR, SyrM, and TtsI). This model does not distinguish the type of interaction (transcriptional or posttranscriptional). It only represents how a specific regulator modulates (positively or negatively) the level of expression of the genes coding for the other regulators.

In previous works we have analysed the relations between the main regulatory proteins involved in the modulation of the expression of the HH103 *nod* regulon. In the presence of genistein, NodD1 induces the expression of TtsI (and consequently that of the T3SS) and SyrM (López-Baena et al., 2008; Pérez-Montaño et al., 2016) through its binding to NB18 and NB19 respectively. SyrM, in turn, activates the expression of *nodD2*, presumably due to its biding to a well-conserved SyrM box located upstream of this gene (Acosta-Jurado et al., 2020). As we show in this work, NodD2 represses the expression of many genes belonging to the *nod* regulon, presumably due to its previously demonstrated repressor effect on *nodD1* expression (MaChado et al., 1998). The nature of this repression (transcriptional or post-transcriptional) remains to be determined. Finally, NolR represses a high number of genes of the *nod* regulon, both because of its repressor effect on *nodD1* but also due to the presence of NolR boxes within the promoters of many of the genes of this regulon (Vinardell et al., 2004b; Acosta-Jurado et al., 2019, **Supplementary Data Sheet 4**), including *syrM* and *ttsI*. In this work we have further investigated these relations by integrating RNA-seq data of individual mutants for each of these regulators with *q*PCR analyses and β-galactosidase assays of individual mutants of these genes targeted with the *lacZ* gene (**Table 3**, **Figure 3**). In general, our data support all these previous observations, although we have also found new relations between some of these regulators previously not reported. The *nodD1* gene is repressed by NolR and NodD2, and it shows autorepression. Repression by NolR might be due to a well conserved NolR box located upstream of *nodD1* (Vinardell et al., 2004b), although this regulatory motif is inversely orientated with respect to *nodD1*. The presence of an inversely orientated NB upstream of *nodD1* might account for its auto-repression in the presence of genistein, as scored by *q*PCR and RNAseq analysis. The fact that the expression of the *nodD1*::*lacZ*Δp-Gm^R^ fusion in the single mutant was not influenced by the presence of genistein (**Figure 3**) might be due to the absence of a functional NodD1 protein in that mutant. The expression of *nodD2* is clearly dependent on NodD1 and enhanced by SyrM, coherently with the presence of a NB upstream of *syrM* and that of a SyrM box in the promoter region of *nodD2*.The repressor effect of NolR on *nodD2* expression might be the result of the repression of the expression of both *nodD1* and *syrM* by NolR. Regarding *nolR*, our β-galactosidase assays indicate that none of the other regulator proteins analysed in this work influence its expression. However, *nolR* shows a clear autorepression, that might be caused by the presence of a NolR box in its upstream region, although this motif is inversely orientated with regard to *nolR* (Vinardell et al., 2004b). In addition, both *q*PCR and RNA-seq analyses suggest a possible positive effect of SyrM in the level of *nolR* transcripts. This fact suggests that SyrM might have a positive effect on *nolR* expression at the posttranscriptional level, although further research would be needed to clarify this issue. In the case of the *syrM* gene, all our results corroborate previous findings that is transcription is dependent on NodD1 through a well-conserved NB (Pérez-Montaño et al., 2016), but also indicates that *syrM* expression is repressed by itself, NodD2, NolR, and surprisingly, by TtsI. The repressor effect of NolR could be due to the presence of a NolR box in the upstream region of *syrM*, and that of NodD2 might be the result of the repressor effect of this protein on *nodD1* expression. However, at present, we do not have clues about how SyrM or TtsI influence *syrM* expression. Finally, the positive and negative effects of NodD1 and NolR on *ttsI* expression can be justified by the presence of a NB and a NolR box in the upstream region of this gene. In addition, *ttsI* is repressed by NodD2and SyrM, but also by itself. Again, the negative effect of NodD2 might be due to its repressor activity on *nodD1*. At this moment, we do not know how SyrM and TtsI repress the expression of *ttsI*. In any case, our data indicate that *ttsI* and *syrM* seems to negatively regulate each other. Also, to our knowledge, this is the first time that TtsI is described as a repressor, in this case of *syrM* and itself., but also of other genes belonging to the *nod* regulon and, to our knowledge, non-related to the T3SS, such as SFHH103_01317 and SFHH103_02323. These results open the possibility that TtsI might act as a regulator not only of the T3SS but also of other processes that remains to be elucidated. **Figure 5** shows the regulatory motifs located in the upstream regions of the five regulatory genes analysed in this work and summarizes the relations we found among those five regulators. This regulatory scheme might be even more complex since there is at least another regulator showing a clear influence in the HH103 *nod* regulon, MucR1 (Acosta-Jurado et al., 2016b), that has not been studied in this work.

Previous works of our group showed that inactivation of different regulatory genes extended the host-range of HH103 to *L. japonicus* and *P. vulgaris*, two legumes in which the wild-type strain is unable to nodulate effectively (Acosta-Jurado et al., 2019; Acosta-Jurado et al., 2020; Jiménez-Guerrero et al., 2020; Fuentes-Romero et al., 2022). In the specific case of *L. japonicus*, the gaining of effective nodulation can be achieved either by inactivation of *ttsI*, which avoids T3SS assembly and T3Es delivery, or that of *nodD2*, *nolR*, or *syrM*, which leads to an overproduction of NFs. The symbiotic performance of mutants in any of the three latter genes was better than that of the *ttsI* mutant (Acosta-Jurado et al., 2019; Acosta-Jurado et al., 2020; Jiménez-Guerrero et al., 2020). In this work we have investigated whether the different combination of two mutations among the four mentioned regulatory genes might affect the symbiotic performance with *L. japonicus* in comparison with the single mutants (**Figure 4** and **Supplementary Figure S1**). All the double mutants tested in this work retained the ability to induce the formation of nitrogen-fixing (pink) nodules, as confirmed by ARA. In the case of the three double mutants harbouring an inactivated copy of *ttsI*, all of them showed worse symbiotic performances (values of PTFW and ARA) than the corresponding single mutants in either *nodD2*, *nolR*, or *syrM*, and similar to that of the *ttsI* single mutant (with the only exception of the double mutant *ttsI nolR*, which gives intermediate values of PTFW and ARA with regard to the single *nolR* and *ttsI* mutants). These results suggest that the gaining of infection ability caused by the absence of a functional T3SS predominates over the effect of the *nodD2*, *nolR* and *syrM* mutations, which most probably is the overproduction of NFs. One possibility to explain this fact would be that the *ttsI* mutation might negatively affect bacterial fitness. However, at least in laboratory conditions, bacterial growth is not affected when the *ttsI* gene is inactivated (López-Baena et al., 2008). However, we have recently found that bacterial motility is negatively affected in the absence of the T3SS (Alías-Villegas et al., 2022), so we may speculate that the T3SS mutant could be affected in root colonization. Alternatively, since T3Es may act suppressing plant immune responses (Jiménez-Guerrero et al., 2022), we cannot exclude the possibility that some T3E might be required along with increased NF production for a better symbiotic performance with *L. japonicus*.

Regarding the combinations of double mutants among the three genes whose inactivation led to overproduction of NFs (*nolD2*, *nolR*, *syrM*), only the double mutant *nolR syrM* exhibited a similar performance to that of the corresponding single mutants. In the rest of the cases, plants inoculated with the double mutants exhibited PTFW and ARA values that were smaller than those of the plants inoculated with the corresponding single mutants. We have also compared the production of NFs by HH103 single and double mutants in the *nodD2*, *nolR* and *syrM* genes. As shown in **Supplementary Data Sheet 8** and **Table 4**, overproduction of NFs was more evident in the single than in the double mutants, and this fact was also true for the only double mutant, *nolR syrM*, whose symbiotic performance was similar to that of the corresponding single mutants.

NF recognition is a key event for the establishment of the symbiosis (Radutoiu et al., 2007; Broghammer et al., 2012; Ghantasala and Choudhury, 2022). As discussed in a previous work (Acosta-Jurado et al., 2019), NFs produced by HH103 and the *Lotus* symbiont *Mesorhizobium loti* R7A are structurally related. Both set of NFs predominantly harbour C16 and C18 saturated or monounsaturated acyl groups and a fucosyl moiety linked to the N-acetyl-glucosamine residue located in the reducing end (Bek et al., 2010; Margaret et al., 2011). However, there are also differences: the presence of carbamoyl substitutions and 4-O-acetyl (or 3-O-acetyl) in *M. loti* NFs and that of 2-O-methyl substitutions in the fucosyl residues of HH103 NFs. The different HH103 single and double mutants in the *nodD2*, *nolR* and *syrM* genes share a set of common NFs that are either overproduced with regard to HH103 or absent in the parental strain (**Table 5**). HH103 is able to induce the formation of white nodules but unable to infect *L. japonicus* roots (Acosta-Jurado et al., 2016a), so the set and amounts of NF produced by this strain are able to trigger nodule organogenesis but not bacterial infection. Our current hypothesis is that some of the NFs overproduced or exclusively produced by the *nodD2*, *nolR* and *syrM* single and double mutants might be essential for triggering the infection of *L. japonicus* roots by *S. fredii* HH103. However, the inactivation of either *nodD2*, *nolR*, and *syrM*, affects the expression not only of genes related to NFs production, but also that of hundreds of other genes. Thus, these regulatory genes affect the production of other bacterial molecular signals such as the repression of the symbiotic T3SSs in the case of *nodD2* and *nolR* or that of EPS in the case of *syrM* (Acosta-Jurado et al., 2016c). Thus, the combination of two mutations among these three regulatory genes might affect differentially the expression of certain genes related with other traits relevant for symbiosis, such as a better capacity of infection or nitrogen-fixation ability, which would explain the observed differences in symbiotic performance among the *nodD2*, *nolR*, and *syrM* different single and double mutants. However, we cannot rule out the possibility that some of the NFs exclusively overproduced by the single mutants, such as some NFs harbouring fucosyl residues instead of methyl-fucosyl moieties (**Table 5**), might be responsible for the better symbiotic performance of these mutants when compared to the double mutants. Clearly, more research is required to clarify this issue.

## Data availability statement

The datasets presented in this study can be found in online repositories. The names of the repository/repositories and accession number(s) can be found in the article/**Supplementary Material**.

## Author contributions

PN-G: Writing – original draft, Conceptualization, Investigation, Methodology, Formal analysis. FF-R: Writing – original draft, Investigation, Methodology, Formal analysis. FM: Investigation, Methodology, Formal analysis, Writing – original draft, Writing – review & editing. IJ-G: Writing – original draft, Investigation, Methodology. CA-V: Writing – original draft, Investigation, Methodology. PA-G: Writing – original draft, Investigation, Methodology. AA: Writing – original draft, Investigation, Methodology. CM: Writing – original draft, Investigation, Methodology. FO: Investigation, Methodology, Writing – review & editing. MR-C: Writing – original draft, Investigation, Methodology. JR-S: Writing – original draft, Conceptualization. FL-B: Funding acquisition, Project administration, Writing – review & editing. JV: Conceptualization, Investigation, Methodology, Formal analysis, Funding acquisition, project administration, Supervision, Visualization, writing – original draft, Writing – review & editing. SJ: Conceptualization, Investigation, Methodology, Formal analysis, Supervision, Visualization, Writing – original draft, Writing – review & editing.
