# Supplementary material for: A complex regulatory network governs the expression of symbiotic genes in Sinorhizobium fredii HH103
Source: Front Plant Sci. 2023 Dec 21;14:1322435. doi: 10.3389/fpls.2023.1322435 (PMC10771577; doi:10.3389/fpls.2023.1322435)
Supplement: Supplementary file 1 [file Table_1.docx]

**A complex regulatory network governs the expression of symbiotic genes in *Sinorhizobium fredii* HH103**

**Pilar Navarro-Gómez^1,2^, Francisco Fuentes-Romero^1^, Francisco Pérez-Montaño^1^, Irene Jiménez-Guerrero^1^, Cynthia Alías-Villegas^1,2^, Paula Ayala-García^1^, Andrés Almozara^1^, Carlos Medina^1^, Francisco-Javier Ollero^1^, Miguel-Ángel Rodríguez-Carvajal^3^, José-Enrique Ruiz-Sainz^1^, Francisco-Javier López-Baena^1^, José-María Vinardell^1,*^, Sebastián Acosta-Jurado^1,2,*^**

**Supplementary Material:**

**Supplementary Table 1.** *S. fredii* HH103 mutant derivatives generated in this work.

**Supplementary Figure S1.** Symbiotic performance of *S. fredii* HH103 and different mutant derivatives with *L. japonicus*. (**A**) Number of pink nodules per plant. (**B**) Number of white nodules per plant. (**C**) Plant-top fresh weight per plant. (**D**) ARA per Leonard jar. Error bars show standard deviations. Each mutant in either *nodD2*, *nolR*, *syrM,* or *ttsI* was compared to its corresponding double mutants, the wild-type strain and the Non-inoculated plants (NI) by using the non-parametric test of Kruskal-Wallis. The p values are: <0.0001, ****; <0.001, ***; <0.01, **; <0.1, *.

**Supplementary Table S1.** *S. fredii* HH103 mutant derivatives generated in this work.

| **Strain (mutations)** | **Recipient strain** | **Plasmid carrying the mutation** |
| --- | --- | --- |
| SVQ770 (Δ*syrM* *nodD2*::*lacZ*Δp-Gm^R^) | HH103 Rif^R^ Δ*syrM* | pMUS789 |
| SVQ786 (*nodD1*:: *lacZ*Δp-Gm^R^) | HH103 Rif^R^ | pMUS534 |
| SVQ787 (*nodD2*:: *lacZ*Δp-Gm^R^) | HH103 Rif^R^ | pMUS789 |
| SVQ788 (*nolR::*Ω *nodD1*::*lacZ*Δp-Gm^R^) | HH103 Rif^R^ *nolR::*Ω | pMUS534 |
| SVQ789 (Rif^R^ Δ*syrM* *nolR*::*lacZ*Δp-Gm^R^) | HH103 Rif^R^ Δ*syrM* | pMUS859 |
| SVQ811 (Δ*syrM* *nodD1*::*lacZ*Δp-Gm^R^) | HH103 Rif^R^ Δ*syrM* | pMUS534 |
| SVQ814 (*nodD1::*Ω *syrM*::*lacZ*Δp-Gm^R^) | HH103 Rif^R^ *nodD1::*Ω | pMUS1234 |
| SVQ817 (Rif^R^ *ttsI::*Ω *nodD1*::*lacZ*Δp-Gm^R^) | HH103 Rif^R^ *ttsI::*Ω | pMUS534 |
| SVQ819 (*nodD2::*Ω *syrM*::*lacZ*Δp-Gm^R^) | HH103 Rif^R^ *nodD2::*Ω | pMUS1234 |
| SVQ828 (*ttsI*::*lacZ*Δp-Gm^R^ *syrM*::pK18*mob*) | HH103 Rif^R^ *ttsI*::*lacZ*Δp-Gm^R^ *syrM*::pK18*mob* | pMUS1492 |
| SVQ836 (*nodD2::*Ω *nodD1*::*lacZ*Δp-Gm^R^) | HH103 Rif^R^ *nodD2::*Ω | pMUS1534 |
| SVQ837 (*nolR::*Ω *nodD2*::*lacZ*Δp-Gm^R^) | HH103 Rif^R^ *nolR::*Ω | pMUS789 |
| SVQ842 (*ttsI::*Ω *syrM*::*lacZ*Δp-Gm^R^) | HH103 Rif^R^ *ttsI::*Ω | pMUS1234 |
| SVQ843 (*nolR::*Ω *syrM*::*lacZ*Δp-Gm^R^) | HH103 Rif^R^ *nolR::*Ω | pMUS1234 |
