# Supplementary figures and images for: A complex regulatory network governs the expression of symbiotic genes in Sinorhizobium fredii HH103
Source: Front Plant Sci. 2023 Dec 21;14:1322435. doi: 10.3389/fpls.2023.1322435 (PMC10771577; doi:10.3389/fpls.2023.1322435)

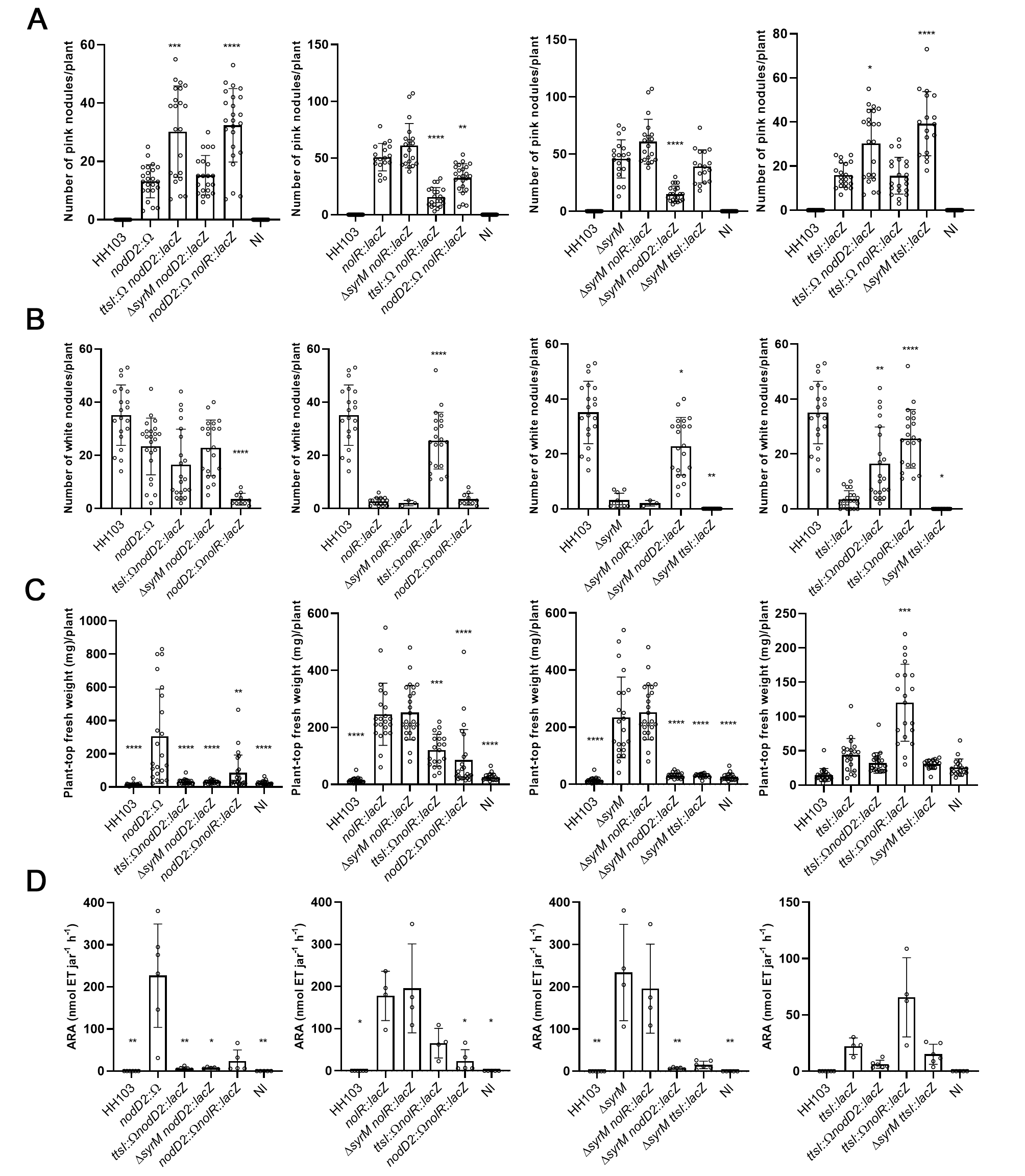

Supplement: Supplementary file 10 [file Image_1.tif]
